# 1889

**Published:** 1889-01

**Authors:** 


### HALL'S JOURNAL OF HEALTH

TRUTH DEMANDS NO SACRIFICE; ERROR CAN MAKE NONE.

VOL. 36. JANUARY, 1889. No. 1.

### 1889.

For the thirty-fifth time since the publication of HALL'S JOURNAL OF HEALTH was undertaken by the late eminent Dr. Hall, it greets its friends and patrons, one and all, with the compliments of the New Year.

In all the past it has been the endeavor of those to whom its conduct has been committed, to make the Journal purely a *family health publication*, as distinguished from a strictly medical or surgical one, giving with each issue information of such safeguards against the contraction of disease and such simple remedies therefor as are found to be within the reach of every one.

The one peculiarity which assigns to it a place occupied by no kindred periodical, is its oft-repeated claim that physical health depends largely upon mental conditions; that both the mental and the physical must be appreciated and understood in order to an intelligible ministration to the afflicted. One of the most glaring deficiencies of what may be termed the old dosing school is, that it undertakes to deal with the physical merely, administering to all ages, capacities, temperaments and conditions in much the same order, without taking it into consideration that a remedy which might be useful in the one case might be just the reverse in the other.

On the other hand, there is a class of healers that utterly ignore the physical in their treatment of the suffering, holding that all forms of disease are mental delusions which a properly directed effort of the mind alone, is able to throw off.

As with most extremes the medium course; the one which advisedly recognizes the dual nature of man; the spiritual no less than the physical, is believed to the true one.

The editorial corps of the Journal includes those who have devoted much time to the study of what is known as Occult Science, which accounts for much that has appeared in its columns in affirmance of the truth of many of those manifestations which transcend the more familiar laws of the natural universe, but it has been our purpose in the past as it will be in the future, to allow nothing to appear, however opposed to popular ideas, which is not susceptible of proof by methods quite convincing to the unprejudiced mind.

With the religious belief and practices of its readers the Journal has nothing whatever to do, but it will not withhold from them a valuable truth out of fear that its conflict with error may disturb their long cherished notions upon any matter affecting their moral status in this world or any other. Even a false light may serve as the pioneer of truth by provoking agitation and inquiry in new directions, leading to right conclusions. Our highest aim is to be of use to our fellow-man, and hold the age somewhat our debtor for having been of it.

